# Multilayer Control of B Cell Activation by the B Cell Antigen Receptor: Following Themes Initiated With Bill Paul

**DOI:** 10.3389/fimmu.2018.00739

**Published:** 2018-04-23

**Authors:** Anthony L. DeFranco

**Affiliations:** Department of Microbiology and Immunology, University of California, San Francisco, San Francisco, CA, United States

**Keywords:** activation, autoimmunity, B cell, B cell antigen receptor, signaling, tolerance, tuning

## Abstract

This article describes the work I did in Bill Paul’s lab as a postdoctoral fellow between 1979 and 1983, and to a lesser extent puts that work in the context of other work on B cell activation and antibody responses that was going on in Bill’s lab at that time and shortly beforehand, including the discovery of interleukin 4. In addition, this work describes the subsequent and continuing work in my own lab following-up on themes I began during my time working directly with Bill. A particular emphasis was on understanding the biochemical mechanisms of signaling by the B cell antigen receptor (BCR) to the interior of the B cell. Some of the studies from my lab related to the regulation of BCR signaling by Lyn are described in relationship to the lymphocyte tuning hypothesis put forth by Grossman and Paul in 1992 and subsequently.

## Studies of B Cell Activation with Bill Paul (1979–1983)

I joined Bill Paul’s research group at NIH in late 1979 as a postdoctoral fellow soon after Bill had focused much of his attention on understanding B cell activation by antigen. I was interested in the biochemical mechanisms by which receptors signaled to the interior of the cell that they had bound their ligand. At this time, before the discovery of the TCR (which was very close on the horizon; Mark Davis joined us a year later), this problem was more readily approachable in B cells than T cells, as anti-Ig reagents were an accepted surrogate for antigen, and there was no equivalent approach in T cells.

Previous to this time, Bill and colleagues had studied how lymphocytes responded to antigen in B cells and T cells and had concluded that membrane Ig on the surface of B cells was a signaling receptor (1), as opposed to serving primarily a binding/focusing function, as proposed by some other investigators. For example, they and others had found that anti-IgM crosslinking antibodies induced vigorous proliferation of mouse B cells. My initial studies with Bill were designed to characterize in more detail the nature of this proliferation. Maureen Howard, who had recently joined Bill’s lab from Australia, used this assay to look for T cell-derived growth factors for B cells (by analogy with IL-2), and she and John Farrar discovered IL-4 by its ability to strongly promote the proliferation of B cells stimulated with a sub-mitogenic concentration of anti-IgM antibodies (2).

The spleen was used as a source of B cells for these experiments, and there was evident heterogeneity in the size of the cells, suggesting that perhaps some of the cells were in the process of activation at the time of isolation. It is now known that splenic marginal zone B cells and B1 B cells have an enlarged pre-activation-like phenotype, in contrast to follicular B cells, which are small, resting lymphocytes. To reduce this heterogeneity, I used Percoll density gradients to isolate a more homogeneous small lymphocyte population of splenic B cells. These cells, comprising 60–70% of splenic B cells, fit the cell biological definition of resting or quiescent cells, as they needed at least 30 h of stimulation before entering S phase and completed the first round of cell division in a synchronous fashion (3, 4). When stimulated with anti-IgM, all of these small, resting B cells exhibited a prolonged period of cell enlargement, corresponding to exit from a quiescent phase (G_0_) and progress through the G_1_ phase of the cell cycle in a process that required continuous stimulation (5). At any time during the first 24 h, removal of anti-IgM caused the cells to stop their enlargement, indicating that progression through early G_1_ phase was dependent on continued B cell antigen receptor (BCR) stimulation (5). This result was somewhat surprising since anti-IgM is very effective at capping membrane IgM molecules and causing their internalization and degradation. However, newly synthesized membrane IgM molecules are present on the cell surface, albeit at low levels, and these studies indicated that their engagement and signaling was required for B cell activation to proceed. After 24 h, progression through S phase, which occurred with about 50% of the stimulated B cells, was now independent of BCR stimulation, consistent with B cells following the cell cycle rules observed in various other mammalian cell types in culture.

Previous work in Bill’s lab had found that anti-IgM failed to induce proliferation of splenic B cells isolated from CBA/N mice or from F_1_ male mice with CBA/N mothers (6), which were subsequently shown to have a loss-of-function mutation in the gene encoding Btk (7), which is located on the X chromosome. Btk is now known to be an important signaling component of the BCR. The mutant locus in CBA/N mice at the time was called *xid*, for X-linked immunodeficiency locus, as these mice had defective antibody responses to polysaccharide antigens. These antigens would induce antibody responses in T cell-deficient mice (nude mice), as would some other antigens that induced responses in *xid* mice. Thus, T cell-deficient mice and *xid* mice were used to characterize antigens into three functionally distinct groupings: T cell-dependent antigens, T-independent type I antigens (those that worked in *xid* mice), and T-independent type 2 antigens (those that did not induce antibody responses in *xid* mice). Based on lack of responsiveness in *xid* mice, anti-IgM most resembled polysaccharide antigens (TI-2 antigens), which made sense in that polysaccharides were thought to be able to effectively crosslink many BCR molecules on the surface of B cells (8) and hence induce strong signaling reactions to stimulate the B cell, a point that was experimentally verified several years later when BCR signaling reactions were identified (9). This analogy only went so far, however, as anti-IgM-stimulated B cells failed to differentiate into antibody-secreting cells *in vitro*, suggesting that additional signals beyond BCR signaling were needed. Along with Maureen Howard and Bill’s pioneering discovery of IL-4, two other groups discovered IL-5 and IL-6, which had distinct effects on B cells *in vitro*. Treatment of anti-IgM-stimulated B cells with highly purified IL-4^+^IL-5^+^IL-6 induced them to terminally differentiate into antibody-secreting cells (10), thereby providing an *in vitro* model mimicking many properties of polysaccharide antigens.

Bill’s interest in using *xid* mice as a tool to uncover aspects of B cell activation in this time period contributed importantly to understanding the differential requirements for antibody responses of polysaccharide antigens vs. other types of antigens and several of my fellow postdoctoral fellows in Bill’s lab were studying antibody responses to pure polysaccharide antigens (11, 12). Remarkably, the understanding that Bill’s lab contributed on this topic would subsequently have relevance to human vaccine design. To make vaccines against several major bacterial pathogens, their cell wall polysaccharides were isolated and used as vaccines. It was subsequently recognized that this type of vaccine was poorly efficacious in very young children (<2 years old), whereas other types of vaccines were effective when used to immunize children several times within the first year of life. Thus, the TI-2 vaccines had limitations that meant that they were unable to prevent some forms of serious disease in young children. The elegant solution was to convert TI-2 antigens to T cell-dependent antigens by attaching an immunogenic protein to them, creating the “conjugate vaccines” (13). Although Bill’s own research efforts were not directed toward this particular development, his earlier studies had laid the conceptual groundwork for the development of conjugate vaccines.

While IL-4, IL-5, and IL-6 could all be made by CD4^+^ T cells, the anti-IgM^+^IL-4^+^IL-5^+^IL-6 model did not seem to fully recapitulate the activity of helper T cells, in part because *xid* B cells could not respond in this system, but made reasonable responses *in vivo* to T cell dependent antigens such as haptenated proteins. At that time, Ron Schwartz’s lab, also in the Laboratory of Immunology at NIH, had become highly proficient at propagating CD4 T cells *in vitro* and could generate clonal cell lines with homogeneous specificity. One of Ron’s postdoctoral fellows, Jonathan Ashwell, now an investigator at NCI, had such T cell clones, and we decided to join forces to try and study how helper T cells and B cells interact to induce T cell-dependent antibody responses. We were able to observe excellent polyclonal proliferation of small resting splenic B cells when we put them together with some of Jon’s clones and added the antigen for that clone. This represented a polyclonal version of earlier experiments published by Singer and colleagues at NIH, who had taken antigen-specific helper T cells, combined them with B cells and achieved *in vitro* activation of the antigen-specific B cells as judged by antibody production. In those studies, to be activated, the B cells had to express the allelic form of class II MHC that was recognized by the helper T cells (14). We thought our system might be able to tease out some aspects of the mechanism by which helper T cells activate B cells, and indeed this was the case, but only after an important issue was resolved first.

Central to these experiments was the issue of whether B cells presented antigen to T cells and if so, what were the functional consequences of that presentation for the two partners in the interaction. Since B cells expressed high levels of class II MHC molecules, it seemed likely that they could present antigen to T cells but did this presentation lead to activation of the T cells or did the recognition of peptide/MHC by the T cell directly send an activation signal to the B cell? With regard to the activation of the T cell, B lymphoma-derived cell lines could present antigen to primary T cells (15), but attempts to demonstrate directly this presentation by primary B cells *in vitro* had often been unsuccessful. We were more focused on the other issue: would the clonal T cells, once activated by adding their antigen, stimulate any B cell or only the B cell presenting antigen to that T cell? If T cell help for B cells was primarily mediated by the cytokines produced upon T cell recognition of antigen, then perhaps the T cell would activate “bystander” B cells. Alternatively, the recognition of peptide/MHC by the T cell might generate a signal only within the antigen-presenting B cell, for example, transmitted by MHC class II molecules upon their engagement by the T cells’ TCR. To address this question, we mixed together with the T cell clones equal numbers of two types of splenic B cells, one expressing the allele of class II MHC molecule that was recognized by the T cell, and the other expressing only non-stimulatory alleles of MHC class II. As the read out for many of these experiments was proliferation of the cell of interest, a common approach was to irradiate the other cells added to the culture, so that they were incapable of incorporating radiolabeled thymidine and hence would not add to the signal. When an unseparated population of spleen cells was irradiated in this way (3,000 R), it retained full activity to activate T cells, so this approach seemed to be valid. For our experiments, we irradiated the T cells and one or the other of the two B cell populations. When we then analyzed proliferation of the two types of B cell, the B cells with the correct MHC class II proliferated when the bystander B cells were irradiated, but the bystander B cells failed to proliferate when the antigen-presenting B cells were irradiated. This turned out to be a misleading answer, as we soon discovered. In discussing this experiment with Bill and Ron Schwartz, Bill was concerned that perhaps the effects seen were due to differential activation of the clonal T cells in the two parallel cultures. While the irradiation procedure seemed to be innocuous, how could we be sure? Bill’s rigorous thought proved to be pivotal. Jon and I went back to the bench and devised an experiment to address Bill’s objection. Now, we did not irradiate either B cell population, but just mixed them together, incubated them with the T cell clone and added the T cell’s antigen. After 24 h incubation, we measured enlargement of the two types of B cells, which we could distinguish by flow cytometry. Now both the antigen-presenting B cells and the bystander B cells became activated to similar extents, indicating that once the T cells were activated, they could activate bystander B cells as well as the B cells that presented antigen to them (16). Of course, the activation of the T cells was MHC restricted, but, at least in this circumstance where there were many T cells present, the means by which T cells activated B cells was not MHC restricted and behaved as expected for cytokines. It was several years later that Randy Noelle at Dartmouth University discovered the molecular mechanism for this activation as being due to CD40L on the surface of the helper T cell (17). T cell recognition of antigen/MHC induces upregulation of CD40L on the T cell, which delivers a critical signal to B cells *via* their CD40. We now know that soluble cytokines such as IL-4 and IL-21 also contribute to the B cell response. The critical nature of CD40L for helper T cell activation of B cells was subsequently verified by the discovery that X-linked hyper-IgM syndrome, in which T cell-dependent antibody responses are highly defective, results from mutations in the gene encoding CD40L (18).

A second outcome of the collaboration between Jon and me was the direct demonstration that primary B cells are very good at presenting antigen to T cells and activating them (19). Whereas macrophages and dendritic cells still presented antigen well following irradiation at 3,000 R, antigen presentation by B cells was very sensitive to irradiation; their antigen presentation function could be maintained by irradiation up to 1,000 R, but above that, their antigen presentation function was abrogated by irradiation-induced apoptosis. Thus, our early experimental design was flawed by a difference in radiosensitivity between small resting B cells and “professional” antigen-presenting cells such as macrophages and dendritic cells. In any case, the direct demonstration of a robust ability of primary B cells to present antigen to T cells and induce their activation, an outcome of this project, was a substantial contribution to immunology at the time, and by challenging Jon and me to improve our experiments, Bill played an essential role in this discovery.

## Biochemical Basis of BCR Signaling (1983–Present)

As I was pursuing the projects described earlier in Bill’s lab at NIH, I was interested in the biochemical basis by which BCR engagement by anti-IgM induced its effects on B cells. In those days, knowledge about receptor signaling mechanisms was limited to a handful of receptors. Given the techniques available at the time, investigating the process in small resting splenic B cells seemed to be a large challenge. Mark Davis was accumulating different mouse B lymphoma-derived cell lines to use in his efforts to isolate and characterize genes that were differentially expressed between B cells and T cells. Mark and Steve Hedrick found that among the cDNAs that were expressed only in T cells, one was found to exhibit DNA rearrangement in the genome of T cells and was then found to encode the TCR β chain, which was the initial cloning of a TCR gene (20). One of the B cell lines accumulated by Mark, WEHI-231, stopped growing when incubated with anti-IgM, demonstrating that it had intact BCR signaling and suggesting that it might be representative of immature B cells contacting self-antigen (3, 4). While in Bill’s lab, I explored the properties of this cell line and decided that when I started my own lab I would use this cell line to study the mechanism of BCR signaling.

Of course how antigen receptors informed B cells and T cells of their encounter with cognate antigen was a fundamental problem and of great interest to many immunologists. Roger Tsien had recently synthesized novel calcium sensing fluorescent dyes that could be loaded into cells and used to measure intracellular free calcium which was widely thought to be important for regulating cell responses and indeed, it turned out that both B cells and T cells stimulated *via* the BCR or TCR exhibited a rapid and robust increase in intracellular free calcium (21). At this time, a novel lipid signaling reaction, involving hydrolysis of phosphatidylinositol 4,5-bisphosphate (PIP_2_) had been recently characterized in a number of different cell types and Gerry Klaus and collaborators then showed that B cells stimulated by the BCR robustly triggered this signaling reaction (22). We confirmed that this was also true of the WEHI-231 cell line and that one of the second messengers released, inositol 1,4,5-trisphosphate could release calcium from intracellular stores present in B cells, as in smooth muscle and other non-lymphoid cell types (23), indicating that hydrolysis of PIP_2_ was likely upstream of the rapid rise in intracellular free calcium seen upon treatment of B cells with anti-IgM. PIP_2_ hydrolysis seemed to be an important aspect of lymphocyte response to antigen as in both B cells and T cells, since antigen stimulation could be mimicked well by incubating them in a calcium ionophore (to raise intracellular free calcium) and a phorbol ester (24), compounds that were unmetabolizable analogs of the other second messenger generated from PIP_2_ hydrolysis, namely diacylglycerol (DG). At the time, DG and phorbol esters were thought to act *via* a small family of protein kinases called protein kinase Cs. Several years later, it was learned that there are additional signaling reactions downstream of DG or phorbol esters, including activation of the Ras GTPases *via* RasGRP. DG activation of RasGRP is a critical pathway in lymphocytes leading to activation of mitogen-activated protein kinases (25, 26).

As appreciation of the likely important role of PIP_2_ hydrolysis as a mediator of BCR and TCR signaling grew, the problem remained, how did these receptors activate this signaling reaction? The answer emerged from investigators studying how cells became malignant. Cancer researchers Tony Hunter, Mike Bishop, and others, had implicated a new form of protein phosphorylation, tyrosine phosphorylation, as central to growth control in multiple situations, including treatment of cells with growth factors and transformation of cells with certain oncogenes. It emerged that the epidermal growth factor receptor’s intracellular domain was a tyrosine kinase and other tyrosine kinases, such as the viral oncogene v-Src and its normal cellular counterpart (c-Src), were intracellular protein tyrosine kinases associated with the plasma membrane. Mike Gold in my lab used recently developed antibodies against phosphotyrosine to demonstrate that stimulation of the BCR induced very rapid tyrosine phosphorylation of a series of different proteins (27), a result that was also reported independently at about the same type by several other groups (28). In parallel, protein tyrosine phosphorylation emerged as a critical early event triggered by the TCR, with major contributions made by Larry Samelson, Andre Veillette, Joe Bolen, Art Weiss, and others (29).

Soon after the discovery that BCR and TCR stimulation induced tyrosine phosphorylation of multiple proteins, it became evident these phosphorylations represented the key proximal signaling events triggered by antigen engagement (27). For example, Mike Gold and others in my lab set about defining the targets of this phosphorylation (28, 30–32), which made it possible to connect tyrosine phosphorylation to other signaling events such as PIP_2_ hydrolysis and calcium elevation.

In addition to identifying some of the signaling molecules that were the targets of this tyrosine phosphorylation, we set about identifying the tyrosine kinases that were activated and understanding how they associated with the BCR. We found that among the earliest phosphorylated proteins were the membrane Ig associated proteins Igα and Igβ (CD79a and CD79b) (33), and moreover, only the Igα and Igβ of engaged BCR complexes became tyrosine phosphorylated, whereas these subunits of unbound BCR complexes did not become tyrosine phosphorylated. These tyrosines are present in a sequence motif also found in TCR signaling chains (CD3 γ, δ, and γ and ζ chain) and in other immune receptors with similar signaling mechanisms, such as activating Fc receptors, as first identified by Reth (34) and now referred to as the immunoreceptor tyrosine-based activation motif (ITAM). Art Weiss’s group discovered a tyrosine kinase, ZAP70, that binds to TCR ζ phosphorylated ITAMs (35) and is required for their signaling. B cells and most other hematopoietic cell types do not express ZAP70, but do express a very similar protein tyrosine kinase, Syk, and a number of groups showed that it plays an analogous role in BCR signaling (36–39).

These observations left open the question of what tyrosine kinase phosphorylates the ITAM tyrosines, and Src-family tyrosine kinases were implicated as performing this role in T cells (29). B cells primarily express three Src-family tyrosine kinases, Lyn, Fyn, and Blk. To address the role of Lyn, my colleague Cliff Lowell and others generated mice with an inactivated Lyn gene (40–42), and Tomohiro Kurosaki inactivated the Lyn gene in a chicken B cell line, DT-40 (37). In the DT-40 cells, Lyn is apparently the predominant Src-family tyrosine kinase, and the deletion of either Syk or Lyn is sufficient to largely cripple BCR signaling, consistent with the concept that these two types of tyrosine kinases must work in concert to mediate BCR signaling. Genetic analysis of B cells from mice deficient in Lyn, Fyn, or Blk demonstrated that these three Src-family tyrosine kinases are redundant for BCR crosslinking-induced phosphorylation of BCR ITAMs and only deletion of all three results in a block in B cell development at the pre-BCR checkpoint (43). Consistent with a redundant function of Lyn, Fyn, and Blk for initiation of BCR signaling, we found that B cells from Lyn-deficient mice had a slower initiation of BCR-induced calcium elevation and tyrosine phosphorylation of cellular proteins, compared with wild type B cells (40, 44).

## The Tunable Lymphocyte Activation Threshold in B Cells: Regulation of mIgM Expression and B Cell Anergy (1992–Present)

In 1992, Zvi Grossman and Bill Paul proposed a hypothesis that the activation threshold for T cell activation was not a constant with regard to the amount of antigen sensed, but rather was “tuned” by the subthreshold antigen receptor signals that the T cell had received in the recent past (45). This tuning was viewed as transient and an adaptive response to the presence of self-antigens that induced frequent but low intensity antigen receptor signals. Numerous subsequent studies described the applicability of this concept to developing and mature T cells in various situations, and moreover indicated that B cells and NK cells also adapt themselves to their degree of sub-activation stimulation through the BCR and NK cell receptors, respectively (46). In this section, I describe how the Grossman and Paul tuning hypothesis may relate to the regulation of BCR signaling, which has been studied by my lab and by many other labs. In this regard, it should be noted that Grossman and Paul distinguished lymphocyte tuning, which is transient, from developmental or differentiative changes in antigen receptor signaling, such as durable differences that are characteristic of double positive thymocytes vs. naïve T cells or of naïve T cells vs. memory T cells.

A remarkable heterogeneity of mature resting B cells is seen in the level of expression of mIgM, which is in contrast to mIgD, which is expressed by all mature resting B cells at a comparable level. Membrane IgD is not expressed in the most immature B cells in bone marrow and spleen, referred to transitional 1 or T1 B cells, whereas it is highly expressed in mature B cells. Chris Goodnow and colleagues found that mIgM was strongly downregulated in the MD4 Ig transgenic B cells when they came from a mouse expressing the corresponding antigen, lysozyme, as a self-antigen (47, 48). In addition, these B cells were profoundly unresponsive to stimulation *in vitro* or *in vivo*, a condition referred to as anergy. Subsequent studies have found that downregulation of mIgM is a function of the degree of self-antigen recognition for a particular B cell specificity and therefore the majority of mature B cells in the periphery exhibit some degree of self-reactivity (49). Studies of BCR signaling indicate that the downregulation of mIgM also decreases signaling observed when mIgM is crosslinked with anti-IgM antibody or when specific antigen is used in a low valency form, e.g., a form that would not induce strong crosslinking of mIgM molecules (47, 48). Thus, mIgM downregulation is a tuning response of B cells to adjust to their level of self-reactivity. Interesting in this regard, low valency antigens induce robust signaling from mIgM, whereas they are poor stimulators of mIgD on the same cells (50). By contrast, mIgD is able to signal vigorously if extensively crosslinked. Thus, the tuning of the antigen responsiveness of B cells that is observed is primarily applicable to self-antigens that are poorly able to crosslink BCRs. This implies that a foreign antigen present in a higher valency form (as would often be the case on a virus particle or a microbial cell surface) would still be able to stimulate vigorously those B cells that have tuned their responsiveness due to some self-reactivity.

While the Grossman and Paul concept of lymphocyte tuning and the concept of B cell anergy both address the result of B cell self-reactivity, the former concept emphasizes the potential of these cells to participate in immune responses, whereas the latter concept focuses attention of the role of tuning in restraining their activation and the maintenance of immune tolerance to self. Despite this difference in outlook, the phenomenon of B cell anergy is best understood by considering it in light of the continuum of mIgM downregulation seen in the normal population of follicular B cells and the concept of lymphocyte tuning. That is to say, B cell anergy should be considered as the property of those B cells with greater degrees of tuning and moreover, the functional defects in anergic B cells also likely represent a continuum from a deeper anergy, as seen in the MD4 anti-lysozyme Ig transgenic mice and as seen in those anti-DNA reactive B cells in which the IgH transgenic 3H9 heavy chain is paired with λ1 light chains (51), to a milder anergy, as seen in B cells from the Ars/A1 transgenic mouse. Milder forms of B cell anergy appear to be very rapidly reversible, probably reflecting primarily changes in localization and activity of signaling regulators within the cell (52), whereas more deeply anergic B cells have a more slowly reversible tuning of their responsiveness, which may in part reflect transcriptional changes in the levels of signaling regulators (47).

## Regulation of BCR Signaling by the Intracellular Protein Tyrosine Kinase Lyn (1995–Present)

Our studies with B cells from Lyn-deficient mice also revealed that Lyn has a second function in B cells in addition to its phosphorylation of BCR ITAMs. While BCR signaling exhibited a short delay in reaching its peak, at later times BCR signaling was elevated in *Lyn^−/−^* B cells compared with wild type B cells. This enhanced BCR signaling results from the loss of Lyn’s unique ability to attenuate BCR signaling by phosphorylation of tyrosines in the cytoplasmic domains of inhibitory receptors, including FcγRIIb and CD22 (44, 53). Phosphorylation of single tyrosines within conserved sequences, called immunoreceptor tyrosine-based inhibitory motifs leads to recruitment to the plasma membrane of inhibitory phosphatases, both SHP-1, which is a protein tyrosine phosphatase that counters BCR signaling at early stages (54), and SHIP-1, a lipid phosphatase that removes the signaling lipid phosphatidylinositol 3,4,5-trisphosphate (PIP_3_), and thereby attenuates a critical branch of the BCR signaling pathway (55). *In vitro* BCR signaling in response to anti-IgM reagents that do not bind to FcγRIIb are similarly enhanced in *Lyn^−/−^* follicular B cells and in *CD22^−/−^* follicular B cells (56), indicating that phosphorylated CD22 provides a tonic inhibition of BCR signaling and that *in vitro*, it is the main inhibitory receptor in B cells downstream of Lyn.

Lyn-deficient mice develop a severe lupus-like autoimmunity, characterized by production of anti-nuclear antibodies (ANAs) starting at about 3 months of age and die of glomerulonephritis after a little more than 1 year of age (53). Deletion of *Lyn* selectively in B cells using Mb1-Cre to induce deletion from a floxed allele of *Lyn* is sufficient to induce ANAs and glomerulonephritis (57), and similar phenotypes are found in *CD22^−/−^* mice (58), *Fc*γ*RIIb^−/−^* mice (59), and mice with a B cell-specific deletion of the gene encoding SHP-1 (54). Lyn is also expressed in dendritic cells, macrophages, and neutrophils and also enables inhibitory receptor function in these cell types. Deletion of *Lyn* selectively in dendritic cells is also sufficient to induce a lupus-like autoimmunity including production of ANAs, but with greater barrier inflammation than is seen in *Lyn^−/−^* mice (60). Thus, the lupus-like autoimmunity seen in *Lyn^−/−^* mice is driven both by Lyn-deficient B cells and by Lyn-deficient dendritic cells. The ANAs produced in *Lyn^−/−^* mice are T cell-dependent and are dependent upon MyD88-dependent signaling in B cells (61). MyD88 is a signaling adaptor that is essential for signaling by most toll-like receptors, including TLR7 and TLR9.

Thus, defects in Lyn, the inhibitory receptors that it phosphorylates in B cells, or the inhibitory phosphatases that become recruited by those inhibitory receptors upon their phosphorylation, all predispose mice to lupus-like autoimmunity, suggesting a critical role for Lyn-mediated attenuation of BCR signaling in preventing autoantibody production to nuclear components. Production of these particular autoantibodies depends on MyD88 signaling in the B cells, both in *Lyn^−/−^* mice and in other mouse models of lupus that have been examined (62, 63). It appears that the nucleic acid present in apoptotic debris can promote activation of DNA or ribonucleoprotein-specific B cells *via* a synergy between exaggerated BCR signaling (resulting from loss of Lyn-mediated inhibitory pathways) and TLR7 or TLR9 signaling in the B cell. In support of this concept, it is clear that TLR7 or TLR9 can greatly enhance B cell responses to virus-like particles that contain ligands for TLR7 or TLR9 (64), or to hapten-carrier conjugates with attached TLR9 ligands (65) and this requires cell-intrinsic TLR signaling in the responding B cell.

Lyn-mediated inhibitory pathways may be one mechanism of tuning of B cells, as described by the Grossman and Paul lymphocyte tuning hypothesis. The downregulation of mIgM cell surface expression is likely to be a major mechanism of B cell tuning, and this function appears to occur normally in Lyn-deficient B cells. Nonetheless, Lyn-mediated inhibitory signaling may be a second important mechanism of tuning of B cells. Although the molecular mechanisms of tuning of T cells are not entirely defined, various studies suggest that upregulation of the inhibitory receptor CD5 is one mechanism of tuning of T cells and recruitment of SHP-1 to active TCRs represents another mechanism (46). Thus, an involvement in B cells of Lyn, CD22 and SHP-1 in tuning would be consistent to what is currently known about the signaling mechanisms of tuning of T cells. Moreover, various studies indicate that anergic B cells exhibit striking attenuation of BCR signaling (66) and moreover this attenuation has the hallmarks of tuning, as it is induced by chronic contact with self-antigen and is rapidly reversible if the self-antigen is removed. Lyn inhibitory pathways are likely to be at least partially responsible for the attenuation of BCR signaling seen in anergic B cells since the maintenance of anergy requires the presence of both SHP-1 and SHIP-1 (52), both of which are largely dependent upon Lyn for their recruitment to inhibitory receptors in the plasma membrane.

## Concluding Thoughts

Bill Paul’s great contributions to the field of immunology came in many spheres and in many ways (1). In this article, I have discussed Bill’s influence on me as I entered the field of immunology and discussed how his theoretical contributions regarding modulation of the strength of antigen receptor signaling has considerable relevance to some of the experimental systems that I have pursued in my own independent laboratory in the years since my time as a postdoctoral fellow in his lab. Clearly, Bill had a long-lasting and positive impact on the field of immunology in many ways, but his roles as a mentor and as a thought leader in developing overriding concepts about how the immune system functions were two of his more important impacts.

## Author Contributions

AD wrote the text and is wholly responsible for the opinions expressed.

## Conflict of Interest Statement

The author declares that the research was conducted in the absence of any commercial or financial relationships that could be construed as a potential conflict of interest. The reviewer SP and handling Editor declared their shared affiliation.
